# The Role of N-Acetyltransferase 8 in Mesenchymal Stem Cell-Based Therapy for Liver Ischemia/Reperfusion Injury in Rats

**DOI:** 10.1371/journal.pone.0103355

**Published:** 2014-07-24

**Authors:** Jinqiu Fu, Haiyan Zhang, Yong Zhuang, Huan Liu, Qing Shi, Dong Li, Xiuli Ju

**Affiliations:** Department of Pediatrics, Qilu Hospital, Shandong University, Jinan, P.R. China; The University of Adelaide, Australia

## Abstract

**Objective:**

To evaluate the impact of mesenchymal stem cells (MSCs) against hepatic I/R injury and explore the role of N-acetyltransferase 8 (NAT8) in the process.

**Methods:**

We investigated the potential of injected MSCs systemically via the tail vein in healing injuried liver of the SD rat model of 70% hepatic I/R injury by measuring the biochemical and pathologic alterations. Subsequently, we evaluated the expression levels of NAT8 by western blotting in vivo. Concurrently, hydrogen peroxide (H_2_O_2_)-induced apoptosis in the human normal liver cell line L02 was performed in vitro to evaluate the protective effects of MSC conditioned medium (MSC-CM) on L02 cells. In addition, we downregulated and upregulated NAT8 expression in L02 cells and induced apoptosis by using H_2_O_2_ to study the protective role of NAT8.

**Results:**

MSCs implantation led to a significant reduced liver enzyme levels, an advanced protection in the histopathological findings of the acutely injured liver and a significantly lower percentage of TUNEL-positive cells, which were increased after I/R injury. In vitro assays, MSC-CM inhibited hepatocyte apoptosis induced by H_2_O_2._ Moreover, overexpression or downregulation of NAT8 prevented or aggravated hepatocyte apoptosis induced by H_2_O_2_, respectively.

**Conclusions:**

MSC transplantation provides support to the I/R-injured liver by inhibiting hepatocellular apoptosis and stimulating NAT8 regeneration.

## Introduction

Ischemia/reperfusion (I/R) injury is responsible for hepatocellular damage during clinical procedures such as liver surgery, hepatic artery ligation, and liver transplantation [1]. The mechanisms of hepatic I/R injury are mainly derived from cellular damage owing to hypoxia and toxic reactive oxygen species generation on reintroduction of O_2_ into ischemic tissues [2], [3]. The current therapeutic strategies, comprising pharmacological, genetic, and surgical interventions, for liver damage restoration are limited by side effects and scientific controversies [4]. Hence, it is particularly urgent to find a novel therapy with good curative effects and few side effects.

Mesenchymal stem cells (MSCs) have been reported to show great promise as a novel strategy for the treatment of I/R injury of the intestine [5], liver [6], and kidney [7]. Several studies have suggested that at least some of the therapeutic beneficial effects of MSCs are mediated by its paracrine factors secreted, such as growth factors, cytokines, and chemokines [8], [9]. Nevertheless, despite the extensive knowledge regarding the paracrine effects of MSCs, little is known about the influence of MSCs on enzyme expression in the liver.

In the current study, we developed a rat model of 70% hepatic I/R injury and demonstrated an improvement in the liver after MSC treatment. Subsequently, we conducted a gene chip analysis, which compared the fold change of hepatic mRNA of hepatic I/R injury rat with that of MSC-treated I/R rat. The result showed that the fold change of N-acetyltransferase 8 (NAT8) was extremely significant (unpublished data). NAT8, as a microsomal enzyme, is exclusively expressed in human kidney and liver, and has been suggested to play an important role in the development and maintenance of the structure and function of kidney and liver [10]. Furthermore, because of the difficulty in developing NAT8 knockout rats, we downregulated and upregulated NAT8 expression in normal human hepatocyte L02 cells and induced apoptosis by using H_2_O_2_ to study the protective role of NAT8.

## Materials and Methods

### Model of Hepatic I/R Injury

Female SD rats weighing about 200 g were purchased from the Experimental Animal Centre of Shandong University and housed in a pathogen-free environment with a 12 h–12 h light-dark cycle. Food and water were available and libitum. All the experimental procedures were approved by the Animal Care and Use Committee of Qilu Hospital and conducted under the guidelines for Animal Care and Use of Shandong University, China. All animals were treated humanely according to the guidelines of the Institutional Animal Care and Use Committee of Qilu Hospital, Shandong university. The approval number of Ethic Committee in Qilu hospital of Shandong university is DWLL-2013-16. Hepatic I/R injury was achieved as previously described [11]. The rats were laparotomized after being anesthetized with intraperitoneal pentobarbital sodium (40 mg/kg) and a sterile pediatric vessel loop was placed around the portal triad for 30 min to induce total hepatic ischemia and mesenteric congestion. After the loop was removed, the livers were reperfused for 6 or 24 h.

3×10^6^ MSCs suspended in 0.5 mL of PBS were injected into MSC group via the tail vein (n = 10) when reperfusion was initiated. For control, the same volume of PBS was transfused into the I/R group via the same route (I/R group, n = 10). The sham-operated animals (sham group, n = 10) underwent same operation. After anesthetizing animals in target time points, serum was collected by sterile cardiac puncture, and the picture and weigh of liver was took. Portions of the liver were placed in 10% neutral buffered formalin, and the remaining liver samples were frozen in liquid nitrogen.

### Umbilical-cord-derived MSC Preparation, Culture, and Identification, and Preparation of MSC conditioned medium

The umbilical cords (UCs) were dissected after thorough washing and the blood vessels were removed. The use of UCs was approved by Ethic Committee in Qilu hospital of Shandong university, and the written informed consent from the donor was provided by department of obstetrics in Qilu hospital of Shandong university. The small fragments(1 mm^3^ to 2 mm^3^) were cut and placed in dishes with L-DMEM, containing 10% FBS, 100 U/mL penicillin, and 100 µg/mL streptomycin for culture at 37°C with 5% CO_2_. After 7 to 12 days, the small tissue pieces were removed from the culture and the adherent fibroblast-like cells were cultured to confluence. The cells were then trypsinized and passaged at 1×10^4^ cells/cm^2^ in the medium described earlier. The cells were used after five to seven passages.

Fifth- to seventh-passage cells were stained with either fluorescein-isothiocyanate-conjugated or phycoerythrin-conjugated monoclonal antibodies in 100 µL of phosphate buffer for 15 min at room temperature, as suggested by the manufacturer. The antibodies used were against human antigens CD29, CD31, CD34, CD45, CD44, CD73, CD90, CD105, and CD271 (BD Pharmingen, United States) compared with corresponding isotype control antibodies. The cells were analyzed by a flow cytometry system (Guava easyCyte8HT, EMD Millipore, Billerica, MA), and the data were examined with Guava Incyte (EMD Millipore).

To obtain human MSC conditioned medium (MSC-CM), the cells were cultured in RPMI-1640 supplemented with penicillin (100 U/mL), streptomycin (100 µg/mL), and 10% FBS of a selected batch. We cultured MSCs to70% to 80% confluence and collected the medium at approximately 48 h after refreshing it. The conditioned medium was subsequently centrifuged for 800×g for 5 min to remove detached MSCs and stored at −80°C until further use.

### Cell Culture and Treatment in vitro

Human hepatic L02 cells were cultured at 37°C and 5% CO_2_ in RPMI-1640 medium containing 20% FBS, 100 U/mL penicillin, 100 µg/mL streptomycin, and 10 µg/mL insulin, which were previously published [12]. The written informed consent for the collection and generation of L02 cells approved by ethics committee of Shanghai was provided by Cell bank of Shanghai, China. Subsequently, the cells were stimulated with 1 mM H2O2 for 3 h to test the apoptotic effects. This protocol resulted in the creation of three groups: a normal control group (L02), a H_2_O_2_ treatment group (L02+ H_2_O_2_), and a H_2_O_2_ and MSC-CM treatment group (L02+ H_2_O_2_+ CM). The cells of the L02 group were normally incubated, those of the L02+ H_2_O_2_ group were incubated for 3 h with medium containing 1 mM H_2_O_2_, and those of the L02+ H_2_O_2_+ CM group were incubated in medium with 20% MSC-CM and 1 mM H_2_O_2_ for 3 h. Five or more independent cell experiments were replicated. The concentration of H_2_O_2_ (1 mM) and the time point (3 h) applied were determined by preliminary experiments.

### Cell Transfection and siRNA Treatment

To study the effects of the overexpression and downregulation of NAT8, pcDNA3.1-NAT8 and two pairs of siRNA sequences targeting NAT8 were designed and synthesized (Genepharma Co., Ltd., Shanghai, China). The cells were seeded into 6-well plates and cultured to 30% and 60% to 70% confluence for siRNA transfection and plasmid transfection, respectively, which were carried out the next day. Transfection was performed by using Lipofectamine 2000 (Invitrogen, Carlsbad, CA), according to the manufacturer’s instruction, and the culture medium was replaced after 6 h of incubation. After 72 h of transfection, the cells were counted and subjected to cell assay and Western blot analysis. The untransfected cells were considered to be blank control, and the cells transfected with scrambled siRNA or pcDNA3.1 (empty vector) were considered as negative control.

### Biochemical Assays

The expression of serum alanine aminotransferase (ALT), aspartate aminotransferase (AST), and lactate dehydrogenase (LDH) were measured by using a Synchron LX20 system (Beckman Coulter, Fullerton, CA) and expressed in international units per liter (Clinical Laboratory Services, Qilu Hospital Affiliated to Shandong University).

### Position of DAPI-labeled MSCs in the Experimental Rats

To detect the direction of the injected DAPI-labeled MSCs in the experimental rats, the animals were sacrificed at 6 and 24 h after reperfusion. The fresh tissues were cut into small tissue blocks (3×4×5 mm^3^) and then embedded in Tissue-Tek (optimal cutting temperature compound, Sakura Finetek, Torrance, USA) in the usual manner. Thin frozen sections (5 µm) of the tissues were prepared and observed under inverted fluorescence microscope.

### Histological Examination

The liver sample in 10% neutral buffered formalin was cut into small tissue blocks (3×4×5 mm^3^) and then embedded in paraffin in the usual way. The paraffin sections (5 µm thick) were cut and prepared for following examination, among which one section was stained for histological examination, one was employed for the detection of apoptosis and the other was collected for immunohistochemical staining. The histological examination was performed after hematoxylin and eosin (H&E) staining for conventional morphological evaluation.

### In situ Detection of Apoptosis in the Liver

By employing the above-mentioned paraffin section, apoptosis was determined by in situ detection of DNA fragmentation using terminal deoxynucleotidyl transferase-mediated 29-deoxyuridine 59-triphosphate nick end labeling (TUNEL) assay with the in situ apoptosis detection kit (TACS; Trevigen Inc, Gaithersburg, MD, USA), according to the manufacturer’s instruction. 5 section were examined in each group. In each section, four fields were selected for examination. TUNEL-positive cells were counted and expressed as number of cells per mm^2^ in the tissue section.

### Immunohistochemistry Examination

Immunohistochemical staining for NAT8 was performed with the streptavidin/peroxidase method. In brief, The slides were incubated with the primary specific antibodies against NAT8 (Thermo Scientific, Rockford, USA) and treated with secondary antibody and then mounted with neutral balsam after counterstaining with hematoxylin.

### Apoptosis Detection by Flow Cytometry

Apoptosis was also evaluated by flow cytometry with Annexin V/propidium iodide (PI) double staining (invitrogen, Eugene, Oregon, USA), according to the manufacturer’s instructions. The analyses were performed by using a guava easyCyte 8HT flow cytometer (Millipore, Billerica, MA).

### Western Blot Analysis

Equal amounts of protein (60 µg) from each sample were separated by 12% SDS-PAGE and transferred to PVDF membranes (Millipore, Boston, MA). The membranes were immunoblotted overnight at 4°C with primary antibodies against rat Bax, Bcl-2, NAT8, and GAPDH (Thermo Scientific, Rockford, USA). Following three 5 mins washings, the membranes were incubated with HRP-conjugated secondary antibody (ZSGB-BIO, Beijing, China) for 1 h at room temperature, washed and visualized by employing enhanced chemiluminescence (ECL; Millipore, Billerica, MA) and recorded by using Kodak films.

### Statistical Analysis

The data are expressed as means ± SEM. For single pairwise comparison of normally distributed samples, a two-tailed *t*-test was used. For multiple comparisons of normally distributed data, one-way ANOVA with Tukey-Kramer posthoc analysis was employed. For histologic analysis and for samples that were not normally distributed, Mann-Whitney U test was applied. For multiple independent groups, Kruskal-Wallis nonparametric comparison was used. For all the analyses, P<0.05 was considered as statistically significant. All statistical analyses were conducted using the Statistical Program for Social Sciences 13.0 software program (SPSS Inc., Chicago, IL).

## Results

### Characterization of MSCs

**Figure 1:**
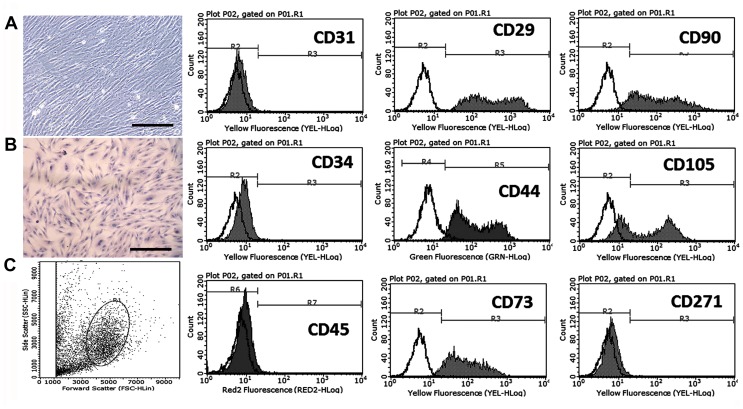
Characterization of UC-derived MSCs. A. Bright-field image. B. H&E staining image. C. Immunophenotype of MSCs at passage 5 by flow cytometry. Scale bars represent 200 µm.

After several passages, the adherent cells from UC could form a monolayer of typical fibroblastic and plastic-adherent cells (Fig. 1A). H&E staining showed spindle-shaped cells (Fig. 1B), and flow cytometry results demonstrated that the UC-derived cells shared most of their immunophenotypes with MSCs, including positive stromal markers expression (CD29, CD44, CD73, CD90, and CD105) and negative hematopoietic marker expression (CD34 and CD45), endothelial cell marker CD31, and differentiated activated effector cell marker CD271 (Fig. 1C). These results indicated that the cells were undifferentiated and had stem cell characteristics.

### MSCs Ameliorate Hepatocellular Damage after Hepatic I/R Injury

The liver injury was assessed by measuring the extracellular release of the hepatic enzymes AST, ALT, and LDH in the serum of the rat model of hepatic I/R injury. As reported earlier [4], these enzymes associated with hepatocellular damage increased in the early stage from 2 h to 6 h after reperfusion and in the delayed phase usually at 24 h after reperfusion. Accordingly, all the experiments in this study were performed at 6 and 24 h after reperfusion.

**Figure 2:**
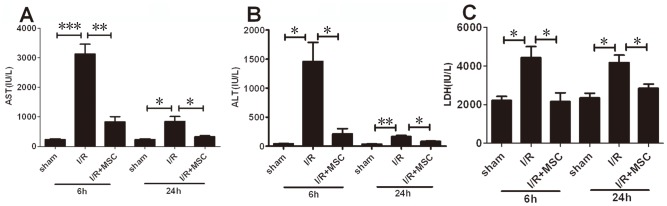
MSCs improved serum biochemical levels in rat with I/R injury. As markers for hepatic injury, serum AST (A), ALT (B), and LDH (C) levels were determined. All values were expressed as mean ± SEM. n = 5 independent experiments. *P<0.05, **P<0.01, ***P<0.001.

The serum AST, ALT, and LDH levels were significantly increased following I/R injury at both 6 and 24 h after operation. At 6 h, the change was approximately 10 folds, as observed in the previous study [6]. However, treatment with MSCs significantly lowered the elevated levels of AST, ALT, and LDH (Fig. 2).

### Microscopic Imaging of the Liver after MSC Transplantation

**Figure 3:**
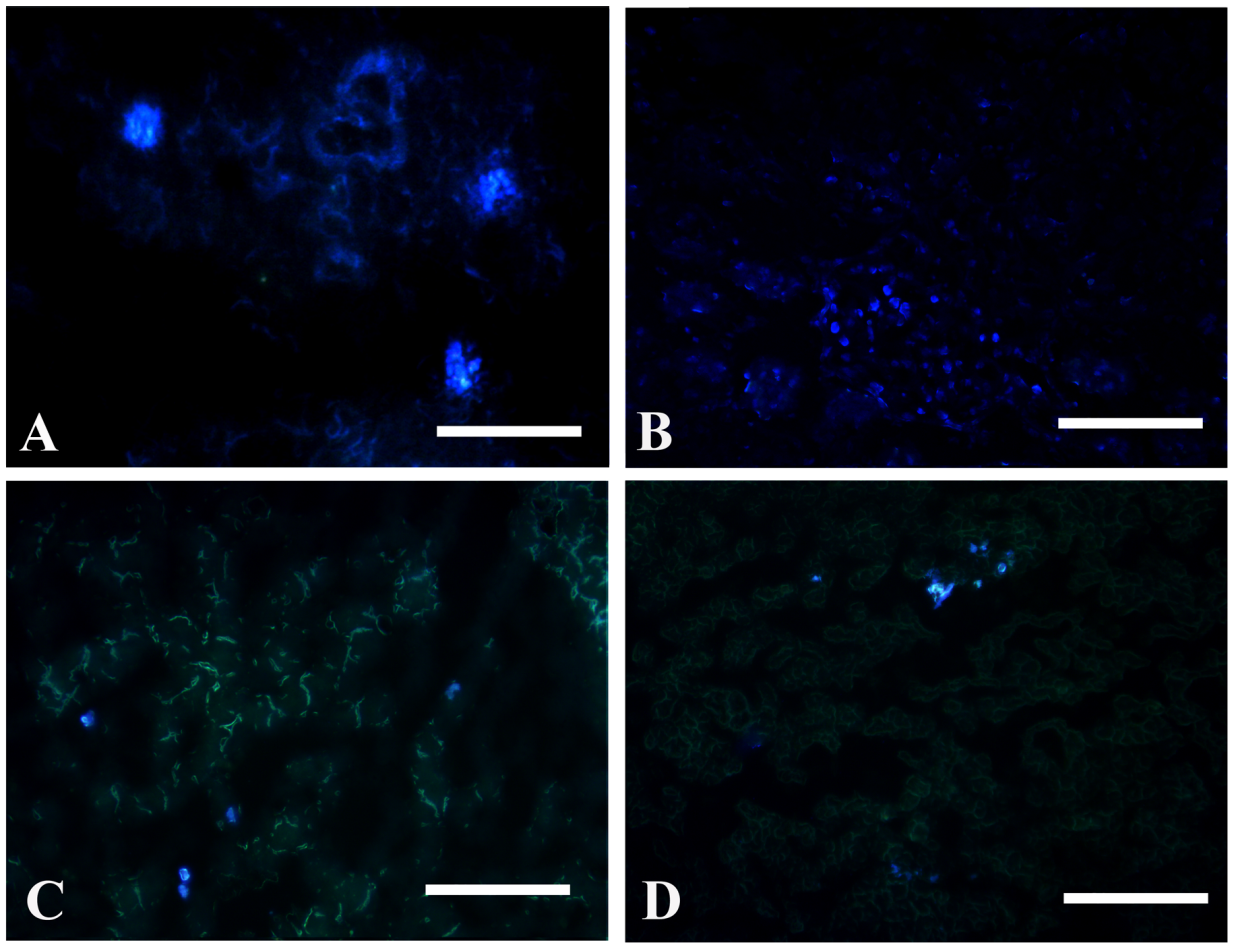
Distribution of DAPI-positive MSCs in the tissues of rat. The frozen sections of lung (A), kidney (B), liver (C), and spleen (D) were observed by fluorescence microscopy. Scale bars represent 100 µm.

To explore the location of MSCs after transplantation, we labeled MSCs with DAPI and injected them into the rat via the tail vein. The DAPI-positive MSCs remained in the lung (Fig. 3A), kidney (Fig. 3B), liver (Fig. 3C), and spleen (Fig. 3D) at 24 h after transplantation. Furthermore, most of the DAPI-positive MSCs were distributed in the lung, suggesting that MSCs may play a more important role in acute lung injury. In addition, DAPI-positive MSCs were noted in the portal triad and interlobular connective tissue of the liver.

### MSCs Promote Hepatic Regeneration after Hepatic I/R Injury

**Figure 4:**
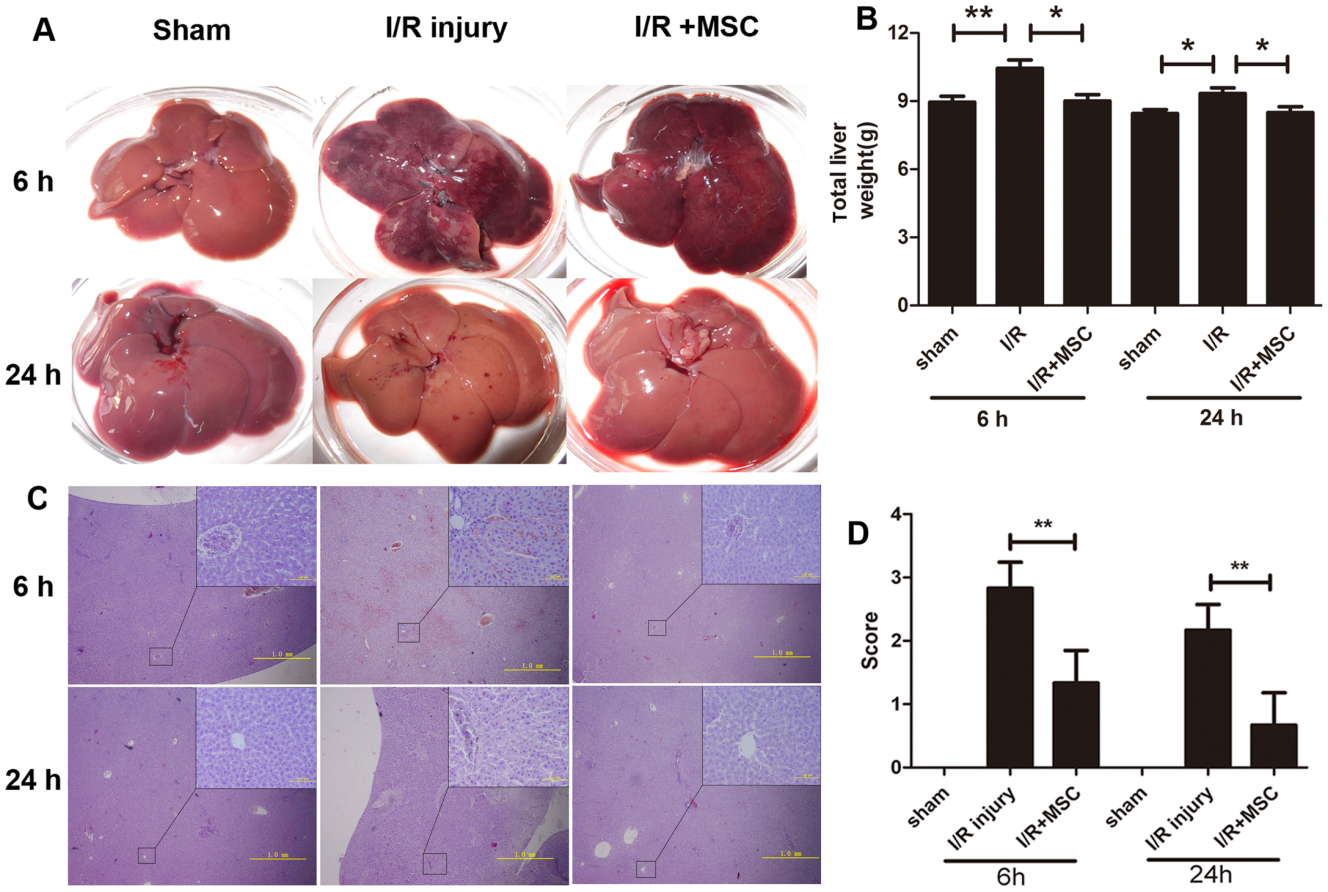
MSCs promoted the regeneration of the liver in rat with I/R injury. A. Appearance examination of livers from the three groups. B. The weight of livers from the three groups. The data are represented as mean ± SEM. n = 6 independent livers. *P<0.05, **P<0.01. C. H&E staining of the liver sections from the three groups. Low magnification (bottom left), scale bars represent 1mm; High magnification (upper right), scale bars represent 100 µm. D. The mean pathological score of the liver sections. All values were expressed as mean ± SEM. n = 5 independent sections. **P<0.01.

To investigate the type and scope of hepatocyte injury, histologic sections of the liver were stained with H&E and scored by two pathologists who were blinded to the experimental groups. The degree of injury was expressed as the mean of 12 different fields within each slide classified on a scale from 0 to 3(normal, 0; mild, 1; moderate, 2; and severe, 3). The apparent changes induced by I/R were examined, including diffuse congestion at 6 h and small focal necrosis at 24 h. A significant reduction of congestion at 6 h and necrosis at 24 h was detected in the liver tissues of animals receiving MSC treatment (figure 4A). The weight of liver in the MSC group was significantly decreased after reperfusion compared with I/R group, as the sham group (figure 4B). At low magnification (figure 4C, bottom left), diffuse congestion and edema at 6 h and small focal necrosis at 24 h became evident after I/R injury, and MSC improved these phenomena. Histological examination displayed that the hepatic I/R led to pathologic changes, including portal inflammation, cytoplasmic vacuolation, apoptotic body production, and hepatocellular necrosis at 6 and 24 h of reperfusion. However, a marked reduction in necrosis was observed in the liver tissues of MSC+I/R group. (Fig. 4C, upper right). Based on the histologic classification, the mean injury score for the MSC+I/R group was significantly lower, when compared with that for the I/R group at 6 and 24 h after reperfusion (Fig. 4D).

### Apoptotic Changes in the Liver after MSC Transplantation

**Figure 5:**
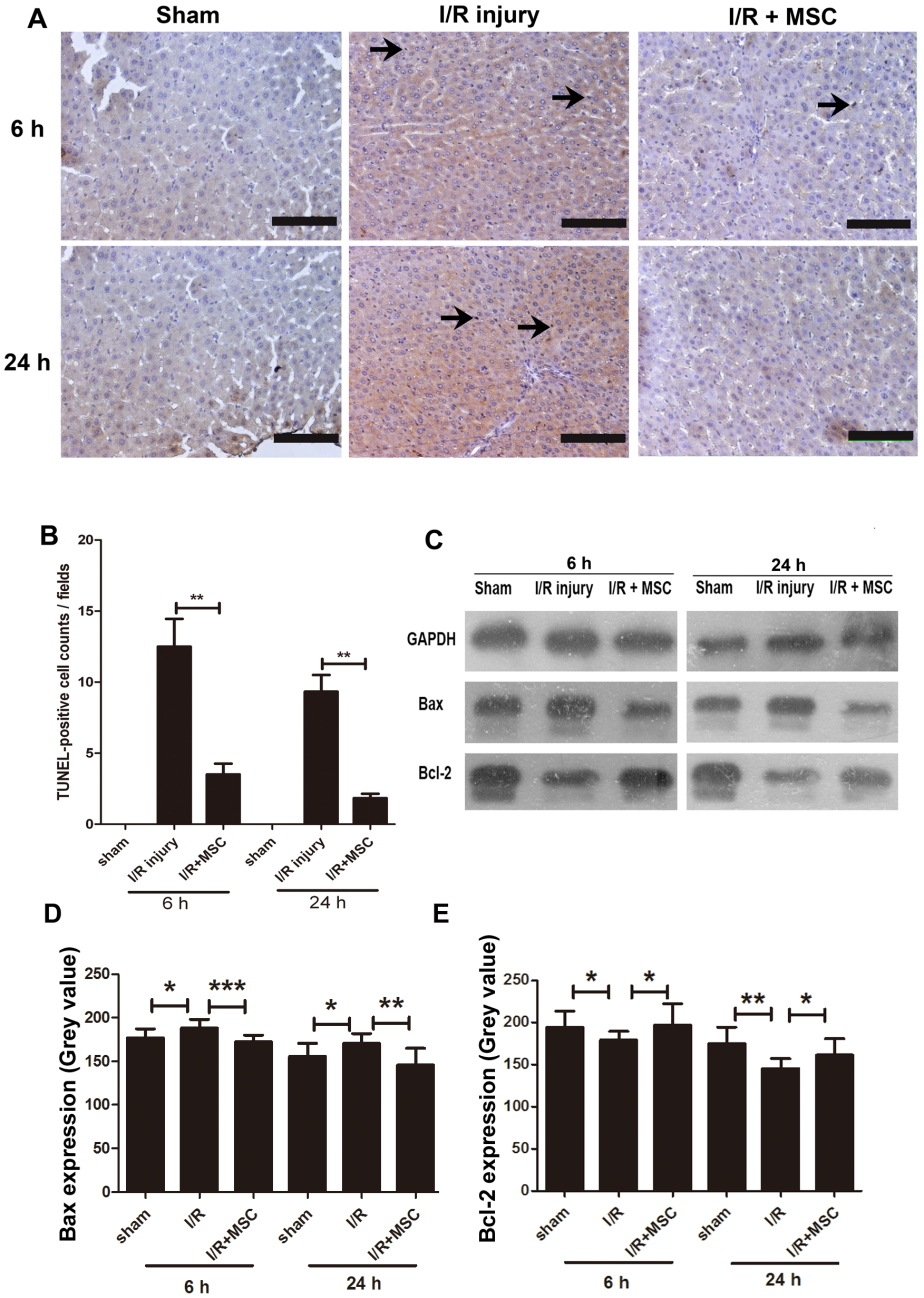
MSCs reduced the apoptosis of liver in rat with I/R injury. A. TUNEL staining of the liver sections from the three groups. Scale bars represent 100 µm. The arrows show TUNEL-positive hepatocyte nuclei. B. Quantification of TUNEL-positive hepatocyte nuclei. n = 5 independent sections. **P<0.01. C. The expression of apoptosis-associated proteins determined by Western blot analysis. D and E. The quantified histogram of Western blot images. *P<0.05, **P<0.01, ***P<0.001.

To further elucidate the pathological changes of I/R and the possible mechanism of MSCs against I/R injury, the liver tissues were subjected to TUNEL assay for apoptosis detection. The TUNEL-positive hepatocytes were mainly localized in the centrilobular region in the I/R group, and apoptosis was clearly detected at 6 h after reperfusion. The systemic infusion of MSCs significantly reduced the number of apoptotic cells in the liver tissues at 6 and 24 h after treatment (Fig. 5A). Quantitative analysis of the extent of hepatocyte apoptosis showed that MSCs significantly reduced I/R-induced liver cell apoptosis, when compared with the I/R group (Fig. 5B). In addition, the apoptotic change in the liver was well correlated with the profiles of the liver enzymes.

To confirm the apoptotic change among the three groups examined, the expression levels of a pro-apoptotic protein Bax and an anti-apoptotic protein Bcl-2 were evaluated by Western blot analysis. Bax expression (Fig. 5C, D) was significantly increased in the I/R group, which was low in the sham control group. However, Bax expression was significantly lower in the MSC group compared to that in the I/R group. On the other hand, Bcl-2 expression (Fig. 5C, E) was significantly decreased in the I/R group compared to that in the sham group, whereas it was significantly higher in the MSC group compared to that in the I/R group.

### NAT8 Participates in MSC-mediated Hepatic Regeneration after I/R Damage in vivo

**Figure 6:**
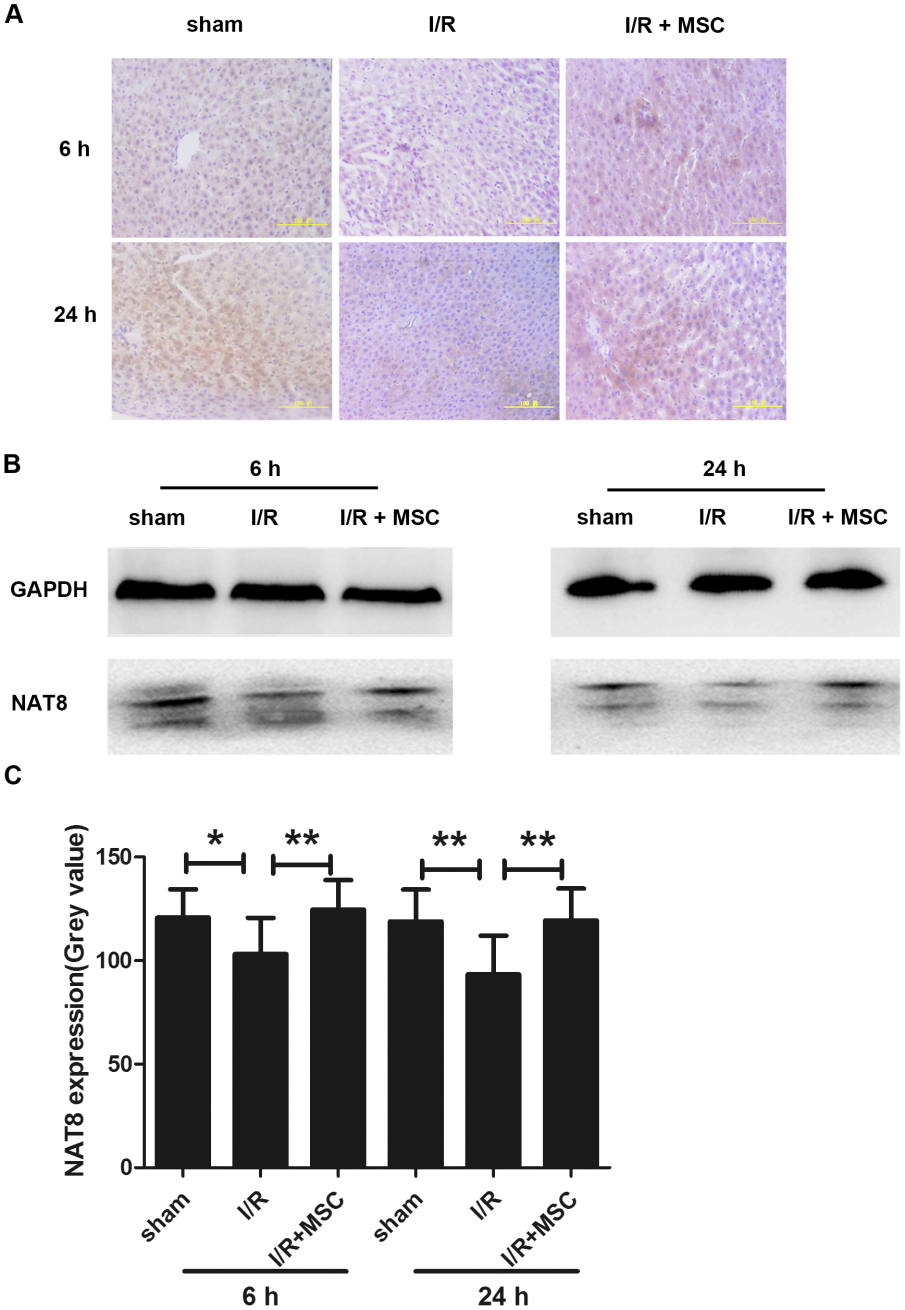
NAT8 participation in MSC-mediated hepatic regeneration after I/R damage. A. Immunohistochemical staining of liver specimens is shown, in which antibodies to NAT8 were used. B. Western blot analysis shows that the NAT8 level was decreased after I/R injury, and MSC treatment resulted in the restoration of the protein level at 6 and 24 h. C. The quantified histogram of Western blot images. *P<0.05, **P<0.01.

To determine the importance of NAT8 in MSCs’ protective mechanisms and I/R-induced hepatic injury, we evaluated the NAT8 expression levels by immunohistochemical analysis and Western blot analysis. Immunohistochemical analysis showed that the NAT8 protein was differently immunostained in the liver cells of three groups (Fig. 6A). As shown in Fig. 6B and C, the level of NAT8 decreased after I/R injury, but was restored following MSC treatment.

### Effects of NAT8-siRNA673, NAT8-siRNA173, and pcDNA3.1-NAT8 on Expression of NAT8

**Figure 7:**
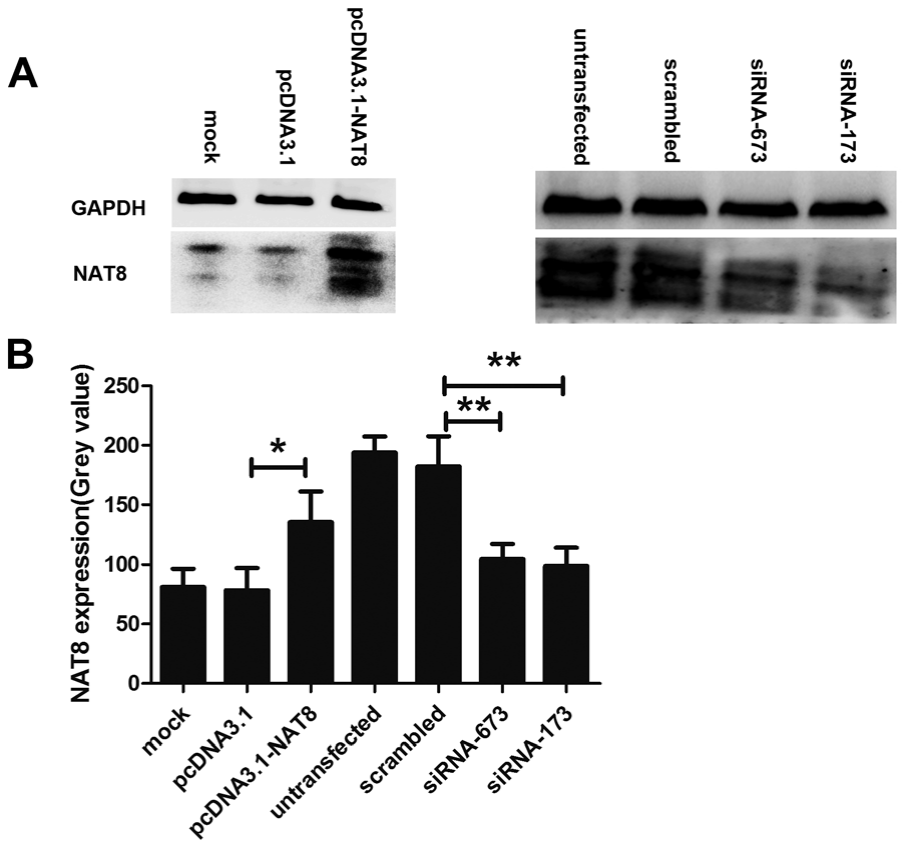
Effects of pcDNA3.1-NAT8, NAT8-siRNA673, and NAT8-siRNA173 on NAT8 expression. A. Western blot analysis shows that pcDNA3.1-NAT8 significantly upregulated NAT8 expression. On the other hand, NAT8-siRNA673 and NAT8-siRNA173 could significantly downregulate NAT8 expression in the L02 cells respectively compared to scrambled siRNA, of which, NAT8-siRNA173 showed more significant RNA inhibiting effect. B. The quantified histogram of Western blot images. *P<0.05, **P<0.01.

As shown in Fig. 7, two pairs of siRNAs and pcDNA3.1-NAT8 targeting NAT8 were employed for apoptosis analysis in normal human hepatocyte line, L02 cells. The results of Western blot analysis demonstrated that NAT8-siRNA673 and NAT8-siRNA173 could significantly downregulate NAT8 expression in the L02 cells respectively compared to scrambled siRNA, of which, NAT8-siRNA173 showed more significant RNA inhibiting effect (Fig. 7A, B, right). On the other hand, pcDNA3.1-NAT8 significantly upregulated NAT8 expression, whereas the transfection reagent and the vector pcDNA3.1 did not significantly affect NAT8 expression (Fig. 7A, B, left). Therefore, NAT8-siRNA673, NAT8-siRNA173, and pcDNA3.1-NAT8 were selected to further study the functions of NAT8 in vitro.

### Effect of NAT8 Expression and MSC-CM on H_2_O_2_-induced Apoptosis of L02 Cells

**Figure 8:**
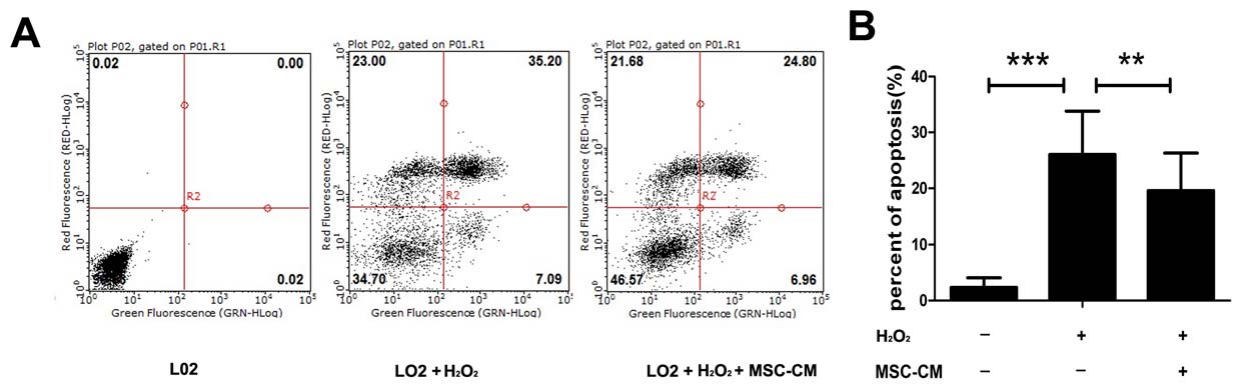
Inhibitory effect of MSC-CM on H_2_O_2_-induced apoptosis of L02 cells. A. Typical protective effect of MSC-CM on the apoptosis of L02 cells apoptosis induced by H_2_O_2_ examinedobserved by Annexin V/PI double staining and flow cytometryic analyses. B. the percentage of apoptotic L02 cells among the total cells. All values were expressed as mean ± SEM of five independent experiments. **P<0.01, ***P<0.001.

To quantitatively determine the effects of MSC-CM on H_2_O_2_-stimulated cell death, we performed Annexin V/PI flow cytometry (Fig. 8A and B) to demonstrate that the MSC-CM could prevent L02 cell apoptosis induced by H_2_O_2_. As shown in Fig. 8A and B, a significant decrease in the percent of apoptosis was observed with 20% MSC-CM supplementation, when compared with that found with 100% RPMI-1640 supplementation at 3 h after H_2_O_2_ injury.

**Figure 9:**
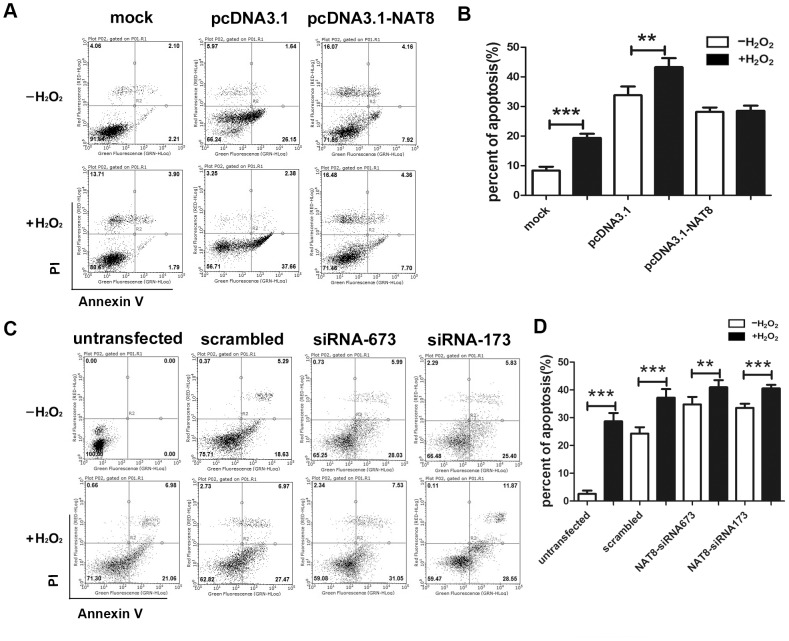
L02 cells with NAT8 overexpression exhibited an increased capacity to resist H_2_O_2_-induced apoptosis. (A and C) Typical effect of NAT8 overexpression and downregulation on H_2_O_2_-induced apoptosis of L02 cells. (B and D) the percentage of apoptotic L02 cells among the total cells. All values were expressed as mean ± SEM of five independent experiments. **P<0.01, ***P<0.001.

Furthermore, the association between NAT8 and L02 cell apoptosis was also explored by using Annexin V/PI flow cytometry. The results (Fig. 9C, D) demonstrated that the percent of apoptosis of NAT8-siRNA673 L02 and NAT8-siRNA173 L02 cells was significantly higher than that of the normal L02 cells at 3 h after H_2_O_2_ injury. In contrast, L02 cells transfected with pcDNA3.1-NAT8 exhibited an increased capacity to resist apoptosis resulting from H_2_O_2_ injury at 3 h. However, no statistically significant differences in the percent of apoptosis were detected between pcDNA3.1-NAT8 L02 group and pcDNA3.1-NAT8 L02+H_2_O_2_ group at 3 h after H_2_O_2_ injury (Fig. 9A, B).

## Discussion

The present study demonstrated that MSCs transplantation was a powerful protective theropy against hepatic I/R injury by promoting the regeneration of the liver, which was noted to be associated with the restoration of liver enzyme upregulation and histopathological changes after hepatic I/R. Furthermore, NAT8 was implicated in rendering protection against hepatocellular injury.

The rat model of total hepatic I/R with intestinal congestion used in the current study differed from other models involving isolated lobar hepatic ischemia. Intestinal congestion and portal outflow obstruction are unavoidable during liver transplantation [13]. To more closely mimic the setting of liver transplantation, in the present study, we clamped the hepatic artery, portal vein, and bile duct to induce total hepatic ischemia and mesenteric congestion. It has been reported that hepatic I/R injury has a biphasic pattern consisting of an early stage and a delayed phase. The early stage appears at 2 h to 6 h after reperfusion with hepatocellular damage, while, the delayed phase usually occurs at 24 h after reperfusion, which leads to a massive infiltration of neutrophils [14]. For this reason, we carried out the entire experiment at the time points of 6 and 24 h after reperfusion. Our results were consistent with the previous studies [6], [12], [15], and the MSCs were viable and functional at 6 and 24 h after transplantation.

Cell therapy as a prominent tool has currently recently been applied in regenerative medicine, given their multipotency, low immunogenicity, self-renewal, and amenability to ex vivo expansion. Previous studies have revealed the protective effects of MSCs on ischemic animal models of cerebral infarction [16], myocardial infarction [17], renal I/R injury [18], and even hepatic I/R injury [6], [12], [15]. However, the mechanism of the beneficial effects of MSCs remains unclear. In the current study, the therapeutic potential and the possible mechanism of the beneficial effects of MSCs in an experimental rat model of hepatic I/R injury were further examined.

It must be noted that approximately one-third of the enzymes that have been described till date have not yet been molecularly characterized, and NAT8 may correspond to an already described enzyme [19]. Maria et al. observed that NAT8 is cysteinyl-S-conjugate N-acetyltransferase, the microsomal enzyme that catalyzes the last step of mercapturic acid formation, which is important for the detoxification and excretion of cysteinyl conjugates [20]. Mercapturic acids are synthesized from the loose glutamate and glycine portions and acetylated glutathione-S-transferase (GST). The primary role of GST is to detoxify xenobiotics by catalyzing the nucleophilic attack by glutathione [21]. Hepatocytes contain high levels of GST, and it has been reported that GST is a valuable indicator of hepatocyte injury in transplantation, toxicity, and viral infections [22]. Although the exact function of NAT8 is unknown, it has recently been shown that NAT8 is associated with detoxification pathways and exerts a protective effect towards both elevated blood pressure and the risk of kidney failure [23], [24]. Furthermore, mutations in the NAT8 gene have been found to be associated with chronic kidney disease [25]. Based on the above-mentioned analyses, we hypothesized that NAT8 plays an important role in the oxidative damage of hepatic I/R injury and promotes MSC-induced-hepatic repair after I/R injury. In the present study, gene chip analysis revealed that the NAT8 level was decreased in the I/R rat and recovered by MSC treatment. Moreover, in vitro, NAT8 overexpression was observed to prevent H_2_O_2_-induced apoptosis of human hepatic cells, suggesting that NAT8 is a crucial enzyme in peroxide damage and asserting the above-mentioned hypothesis.

The effects of MSCs could be multiple. We speculated that the paracrine effects of MSCs, led to secrete a lot of cytokines and growth factors, such as IL-6 [26], HGF [27], and VEGF [28], restrained activation of the MAPK/JNK signaling pathway [29], which is crucial for determining the regeneration of the liver after I/R injury, and subsequently increase NAT8 expression. GST can selectively prevent the action of JNK and thus its induction of the MAPK/JNK pathway, resisting apoptosis [30], [31]. As mentioned earlier, GST is catalyzed by NAT8 to form mercapturic acid, which is excreted. When the hepatic cells are damaged by I/R, cellular oxidative stress causes oligomerization of GST followed by a decrease in the level of NAT8 and induction of the MAPK/JNK pathway, resulting in apoptosis [32]. However, the application of MSCs activates the MAPK/ERK1/2 pathway and inactivates the MAPK/JNK pathway. As a result, the level of GST, as a metabolic enzyme, is increased to inhibit the MAPK/JNK pathway, and the NAT8 level is elevated. In the future, numerous studies will be carried out to further examine the accurate role of NAT8 in the therapeutic effect of MSCs against hepatic I/R injury.

In conclusion, the findings of the present study are consistent with those reported in previous research [6], suggesting that MSCs have the potential to alleviate hepatic I/R injuries and enhance the regeneration of the liver, and that MSC-CM plays a protective role on H_2_O_2_-induced hepatocyte apoptosis. Moreover, NAT8 has been observed to be involved in the molecular mechanism of the therapeutic effect of MSCs against hepatic I/R.
